# Pyorrhea Alveolaris

**Published:** 1918-03

**Authors:** Julio Endelman


### PYORRHEA ALVEOLARIS

BY JULIO ENDELMAN, D.D.S.

The term pyorrhea alveolaris is generally accepted as indicative of inflammatory disorders in the investing tissues of the teeth eventually ending in their exfoliation. The true meaning of the term is "flow of pus from the alveolus," and inasmuch as destruction of the peridental membrane and alveolar bone resulting in the loss of the teeth is not always attended by a flow of pus, the term pyorrhea alveolaris is often incorrectly applied. The loosening and actual loss of a tooth is the result of the destruction of practically all of the peridental membrane and adjacent alveolar bone and it thus happens that all pathologic conditions in these structures as well as in the overlying gum tissues of which the prognosis is loss of a tooth by exfoliation have come to be designated under the generic term "pyorrhea alveolaris." Many terms have been suggested as substitutes for pyorrhea alveolaris, namely, phagedenic pericementitis, interstitial pericementitis, Riggs' disease, alveolo-dental pericementitis, interstitial gingivitis, gouty pericementitis, ptyalogenic calcic pericementitis, dento-alveolitis, etc. None of these terms, however, depicts the series of pathologic phenomena more clearly than the generally employed term pyorrhea alveolaris, and consequently no change in nomenclature seems justifiable at this time, at least not in favor of any of the previously mentioned names.

Clinically several forms of pyorrhea alveolaris are distinguishable. In one form the destructive inflammation in the peridental membrane and alveolar process is preceded by the infliction of degrees of irritation to the gingivae by salivary calculi, in another subgingival or serumal calculi are the source of irritation to the structures involved in the inflammatory process. The etiology of the latter form of calcareous deposits is too well known to require reiteration at this time; suffice it to say, therefore, that subgingival deposits do not occur unless the gingivae have been subjected for some time to irritating stimuli and that weak, insufficient or flat approximal contacts, the rough edges of crown bands, of imperfectly inserted fillings or any other form of mechanical irritation, or of chemical irritation such as develops as the result of decomposition of food debris and of the slimy coating next to the gingivae, are among the most prominent predisposing causes. In all true forms of pyorrhea alveolaris the bacterial factor is to be viewed as the exciter of the inflammatory process which causes the loss of peridental membrane and alveolar process and the exfoliation of the teeth.

In the form caused by salivary calculi the clinical phenomena are not typical of pyorrhea alveolaris, and it should be borne in mind that we consider *the presence of pockets alongside the roots of teeth which have developed there consequent upon the destruction of the peridental membrane and the alveolar process by bacterial activity, or by vascular insufficiency leading to atrophy plus bacterial activity as essential phenomena of true pyorrhea alveolaris.* The pathological phenomena accompanying the destruction of the investing tissue by salivary calculi are not characterized by the formation of pockets and if at all present they are shallow, not more than two millimeters of an average in depth. The peridental membrane, alveolar process and overlying gum tissue are destroyed to the level of the salivary deposit as the result of a combination of pressure atrophy and bacterial activity. Consequently in the salivary calculi form of pyorrhea alveolaris—correctly interpreted the process should be termed calcic gingivitis, pericementitis and alveolitis—the formation of pockets does not occur and the degree of suppurative inflammation under the deposit, if there should be any, is not pronounced at any time, pus forming at a very slow rate and is to be seen at the junction of the deposit with the soft tissues or upon the removal of the deposit. Pus is not by any means invariably associated with salivary calculi; in those cases in which it is present the suppurative inflammation, in addition to the phenomena of pressure atrophy are responsible for the destruction of the investing tissues; in those cases in which no evidence of suppuration is to be detected the investing tissues are destroyed probably mainly as the result of vascular interference leading to atrophy. The appearance of the gum tissues after the removal of a salivary calculus is that of an ulcerated surface, the tissues are red, swollen, the inflammation immediately under the deposit being more severe than in areas not immediately in contact with it. They bleed readily and unquestionably are instrumental in the absorption of bacteria and bacterial toxines responsible for the onset of systemic disturbances.

In the form caused by subgingival deposits, the destructive inflammation affects the peridental membrane, alveolar bone and the gingivae, but the gum tissue proper, while the seat of a chronic inflammation, does not disappear or break down as is the case in the form caused by salivary calculi. Following degrees of irritation to the gingivae by food impactions which find lodgment between the teeth in the presence of defective approximal contacts or in the absence of contacts or because of neglect of the teeth by insufficient brushing, etc.; by the rough edges of crown bands or fillings; by salivary calculi; by severe manipulations of ligatures and rubber dam clamps; by improper handling of the toothbrush producing injuries of the gingival margins, an inflammatory process is inaugurated in these tissues (the gingivae) accompanied by the deposition of calcareous masses under the free gingivae. The subgingival deposits thus produced irritate and subsequently bring about an infectious inflammation of the peridental membrane immediately adjacent to them. Through the action of liquefying bacterial toxines the peridental fibres disappear and the infectious inflammation as soon as the crest of the alveolar process is reached enters the bone and there sets up an osteomyelitis with the absorption of the bony lamellae to which it leads. The inflammation again brings about the deposition of subgingival deposits higher upon the root surface and the processes of irritation, infection, inflammation and bone absorption consequent upon osteomyelitis is repeated and so on until the entire peridental membrane and alveolar process are destroyed and the tooth is exfoliated. In any one of the two forms of pyorrhea alveolaris so far described the presence of any systemic disorder in the digestive, respiratory or urinary tract, or any chronic nervous disorder, or any systemic intoxication of whatever origin it might be acts as the means of ''perpetuating'' the disease in the investing tissues. The causes of the disease may be of a local character but the presence of systemic disorders makes it possible for the disease to gain a foothold upon the investing tissues such as to render its eradication a problem beyond the power of available surgical and therapeutic means.

Any systemic disorder which produces alterations in the quantity or quality of the blood such as for instances Bright's disease, diabetes, or the uric acid diathesis, tuberculosis, syphilis, may act as the systemic predisposing causes of pyorrhea alveolaris. These disorders are responsible for the lowering of the defensive forces of the body and this decrease in vital resistance is perhaps more pronounced in the investing tissues of the teeth for, as pointed out by Talbot, the peridental membrane, alveolar bone, gingivae and gums are in the nature of end-organs and consequently are markedly sensitive to circulatory changes. These systemic derangements are to be viewed in the light of predisposing causes. They render possible the infection of the investing tissues by reason of their having established in those structures areas of deceased vital resistance. The bacterial exciters are of course present at all times in the mouth and, therefore, following the slightest break in the continuity of the tissues consequent upon an injury to the gingival structures bacteria gain entrance and the causes which made this possible persisting the peridental membrane and alveolar bone eventually become involved. The foregoing description applies to a group of cases in which treatment by instrumentation is of only temporary value unless it is carried out at frequent enough intervals to prevent the recurring infection from acquiring an impregnable foothold on the tissues. The systemic factor is frequently associated, in etiology, with a series of local conditions, the same as are responsible for the development of purely local forms of gingivitis and pyorrhea—which leads to equivocal conclusions. Under those circumstances frequently the local causes alone are incriminated when as a matter of fact the systemic disorder is frequently just as much at fault. The former set of causes incite the disease, the latter perpetuate it.

In another group of cases local causes are apparently absent or if present are not possible of determination unless the operator carry out a very minute examination of the gingival tissues. The infection in these apparently obscure cases frequently originates in the subgingival cul-de-sac (subgingival trough) and the initial irritation is traceable to the decomposition by putrefaction, fermentation, or both, of the slimy deposits in proximity to the gingival structures. These slimy deposits are particularly evident in badly neglected mouths and in those of people who neglect the toilet of their teeth immediately after meals. These debris consist of a mixture of saliva and food particles. The end-products of this decomposition, whether alkaline or acid, irritate the tissues in the subgingival trough and infection by the common bacterial inhabitants of the mouth soon follows. The infection once becoming established generally persists, notwithstanding the most careful and thorough operative and therapeutic measures. Some investigators incline strongly toward the elimination of the systemic factor, attributing to purely local causes all the phases and characteristics of the disease. That a considerable proportion of cases is of local causation is admitted by practically all investigators, but that all cases are of that origin has not received, and warrantedly so, that unanimous acceptance.—*Pacific Gazette.*

